# 2-Amino-5-methyl­pyridinium 4-nitro­benzoate

**DOI:** 10.1107/S1600536810005301

**Published:** 2010-02-13

**Authors:** Madhukar Hemamalini, Hoong-Kun Fun

**Affiliations:** a X-ray Crystallography Unit, School of Physics, Universiti Sains Malaysia, 11800 USM, Penang, Malaysia

## Abstract

In the title compound, C_6_H_9_N_2_^+^·C_7_H_4_NO_4_^−^, the nitro group of the 4-nitro­benzoate anion is twisted by 6.2 (2)° from the attached ring. In the crystal structure, the cations and anions are linked *via* strong N—H⋯O and weak C—H⋯O hydrogen bonds, forming a three-dimensional network.

## Related literature

For background to the chemistry of substituted pyridines, see: Pozharski *et al.* (1997 ▶); Katritzky *et al.* (1996 ▶); Hemamalini & Fun (2010 ▶). For details of hydrogen bonding, see: Jeffrey & Saenger (1991 ▶); Jeffrey (1997 ▶); Scheiner (1997 ▶). For hydrogen-bond motifs, see: Bernstein *et al.* (1995 ▶). For bond-length data, see: Allen *et al.* (1987 ▶). For the stability of the temperature controller used in the data collection, see: Cosier & Glazer (1986 ▶).
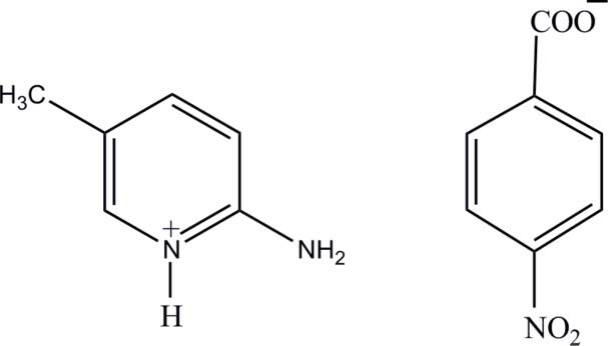


## Experimental

### 

#### Crystal data

C_6_H_9_N_2_^+^·C_7_H_4_NO_4_^−^
*M* *_r_* = 275.26
Monoclinic, 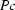

*a* = 13.684 (12) Å
*b* = 4.025 (4) Å
*c* = 12.706 (11) Å
β = 114.94 (2)°
*V* = 634.5 (10) Å^3^
*Z* = 2
Mo *K*α radiation
μ = 0.11 mm^−1^
*T* = 100 K
0.36 × 0.18 × 0.08 mm

#### Data collection

Bruker APEX DUO CCD area-detector diffractometer
Absorption correction: multi-scan (*SADABS*; Bruker, 2009 ▶) *T*_min_ = 0.961, *T*_max_ = 0.991
7053 measured reflections
1854 independent reflections
1283 reflections with *I* > 2σ(*I*)
*R*_int_ = 0.042

#### Refinement

*R*[*F* ^2^ > 2σ(*F* ^2^)] = 0.045
*wR*(*F* ^2^) = 0.116
*S* = 1.06
1854 reflections
222 parameters
2 restraints
H atoms treated by a mixture of independent and constrained refinement
Δρ_max_ = 0.19 e Å^−3^
Δρ_min_ = −0.20 e Å^−3^

### 

Data collection: *APEX2* (Bruker, 2009 ▶); cell refinement: *SAINT* (Bruker, 2009 ▶); data reduction: *SAINT* program(s) used to solve structure: *SHELXTL* (Sheldrick, 2008 ▶); program(s) used to refine structure: *SHELXTL*; molecular graphics: *SHELXTL*; software used to prepare material for publication: *SHELXTL* and *PLATON* (Spek, 2009 ▶).

## Supplementary Material

Crystal structure: contains datablocks global, I. DOI: 10.1107/S1600536810005301/sj2727sup1.cif

Structure factors: contains datablocks I. DOI: 10.1107/S1600536810005301/sj2727Isup2.hkl

Additional supplementary materials:  crystallographic information; 3D view; checkCIF report

## Figures and Tables

**Table 1:** Hydrogen-bond geometry (Å, °)

*D*—H⋯*A*	*D*—H	H⋯*A*	*D*⋯*A*	*D*—H⋯*A*
N1—H1*N*1⋯O3^i^	0.97 (5)	2.47 (4)	3.238 (5)	136 (3)
N1—H1*N*1⋯O4^i^	0.97 (5)	1.77 (5)	2.711 (4)	163 (3)
N2—H1*N*2⋯O4^ii^	0.89 (4)	2.02 (4)	2.905 (4)	179 (5)
N2—H2*N*2⋯O3^i^	0.91 (4)	1.92 (4)	2.804 (5)	165 (4)
C3—H3*A*⋯O1^iii^	0.93 (4)	2.58 (4)	3.514 (6)	176 (3)

Symmetry codes: (i) 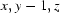; (ii) 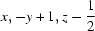; (iii) 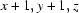.

**Fig. 1.**
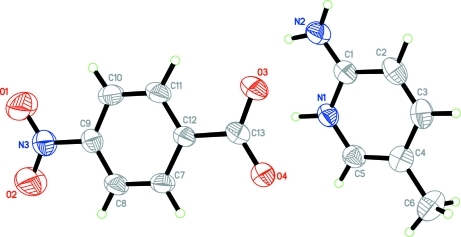
The asymmetric unit of the title compound. Displacement ellipsoids are drawn at the 50% probability level.

**Fig. 2.**
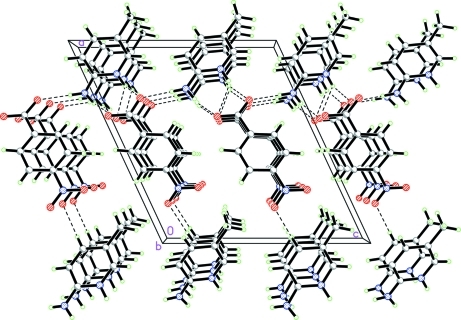
The crystal packing of the title compound, showing hydrogen-bonded (dashed lines) networks.

## supplementary crystallographic information

### Comment

Pyridine and its derivatives play important roles in heterocyclic chemistry (Pozharski *et al.*, 1997; Katritzky *et al.*, 1996). They are often involved in hydrogen-bond interactions (Jeffrey & Saenger, 1991; Jeffrey, 1997; Scheiner, 1997).  We have recently reported the crystal structure of 2-amino-4-methylpyridinium 4-nitrobenzoate (Hemamalini & Fun, 2010).  In a continuation of our studies of pyridinium derivatives, the crystal structure of title compound is presented here.

The asymmetric unit of the title compound (Fig 1), contains a protonated 2-amino-5-methylpyridinium cation and a 4-nitrobenzoate anion. In the 4-nitrobenzoate anion, the nitro group is twisted slightly from the ring with the dihedral angle between O1/O2/N3/C9 and C7–C12 planes being 6.2 (2)°. In the 2-amino-5-methylpyridinium cation, a wide angle (122.0 (3)°) is subtended at the protonated N1 atom.  The 2-amino-5-methylpyridinium cation is planar, with a maximum deviation of 0.015 (4)Å  for atom C2. The bond lengths are normal (Allen *et al.*, 1987).

In the crystal (Fig. 2), the protonated N1 atom and the 2-amino group (N2) are hydrogen-bonded to the carboxylate oxygen atoms (O3 and O4) via a pair of N—H···O hydrogen bonds forming an *R*_2_^2^(8) ring motif (Bernstein *et al.*, 1995).  Bifurcated hydrogen bonds are observed between the carboxylate oxygen atoms (O3 & O4) and the protonated N atom to form a four-membered *R*_1_^2^(4) hydrogen-bonded ring.  The crystal structure is further stabilized by weak C—H···O (Table 1) hydrogen bonds to form a three-dimensional network.

### Experimental

A hot methanol solution (20 ml) of 2-amino-5-methylpyridine (27 mg, Aldrich) and 4-nitrobenzoic acid (42 mg, Merck) were mixed and warmed over a heating magnetic stirrer for a few minutes. The resulting solution was allowed to cool slowly at room temperature and crystals of the title compound appeared after a few days.

### Refinement

The methyl H atoms were positioned geometrically and were refined using a riding model, with *U*_iso_(H) = 1.5*U*_eq_(C). A rotating group model was used for the methyl group. The remaining H atoms were located in a difference map and refined freely [N–H = 0.89 (4)–0.97 (4)Å and C–H = 0.89 (4)–0.98 (4)Å]. In the absence of significant anomalous scattering effects, 1641 Friedel pairs were merged.

### Crystal data

**Table:**

C_6_H_9_N_2_^+^·C_7_H_4_NO_4_^−^	*F*(000) = 288
*M**_r_* = 275.26	*D*_x_ = 1.441 Mg m^−^^3^
Monoclinic, *P**c*	Mo *K*α radiation, λ = 0.71073 Å
Hall symbol: P -2yc	Cell parameters from 1854 reflections
*a* = 13.684 (12) Å	θ = 3.2–28.2°
*b* = 4.025 (4) Å	µ = 0.11 mm^−^^1^
*c* = 12.706 (11) Å	*T* = 100 K
β = 114.94 (2)°	Block, colourless
*V* = 634.5 (10) Å^3^	0.36 × 0.18 × 0.08 mm
*Z* = 2	

### Data collection

**Table:**

Bruker APEX DUO CCD area-detector diffractometer	1854 independent reflections
Radiation source: fine-focus sealed tube	1283 reflections with *I* > 2σ(*I*)
graphite	*R*_int_ = 0.042
φ and ω scans	θ_max_ = 30.0°, θ_min_ = 1.6°
Absorption correction: multi-scan (*SADABS*; Bruker, 2009)	*h* = −18→19
*T*_min_ = 0.961, *T*_max_ = 0.991	*k* = −5→5
7053 measured reflections	*l* = −17→17

### Refinement

**Table:**

Refinement on *F*^2^	Primary atom site location: structure-invariant direct methods
Least-squares matrix: full	Secondary atom site location: difference Fourier map
*R*[*F*^2^ > 2σ(*F*^2^)] = 0.045	Hydrogen site location: inferred from neighbouring sites
*wR*(*F*^2^) = 0.116	H atoms treated by a mixture of independent and constrained refinement
*S* = 1.06	*w* = 1/[σ^2^(*F*_o_^2^) + (0.0604*P*)^2^] where *P* = (*F*_o_^2^ + 2*F*_c_^2^)/3
1854 reflections	(Δ/σ)_max_ < 0.001
222 parameters	Δρ_max_ = 0.19 e Å^−^^3^
2 restraints	Δρ_min_ = −0.20 e Å^−^^3^

### Special details

**Table:**

Experimental. The crystal was placed in the cold stream of an Oxford Cryosystems Cobra open-flow nitrogen cryostat (Cosier & Glazer, 1986) operating at 100.0 (1) k.
Geometry. All s.u.'s (except the s.u. in the dihedral angle between two l.s. planes) are estimated using the full covariance matrix. The cell s.u.'s are taken into account individually in the estimation of s.u.'s in distances, angles and torsion angles; correlations between s.u.'s in cell parameters are only used when they are defined by crystal symmetry. An approximate (isotropic) treatment of cell s.u.'s is used for estimating s.u.'s involving l.s. planes.
Refinement. Refinement of F^2^ against ALL reflections. The weighted R-factor wR and goodness of fit S are based on F^2^, conventional R-factors R are based on F, with F set to zero for negative F^2^. The threshold expression of F^2^ > 2σ(F^2^) is used only for calculating R-factors(gt) etc. and is not relevant to the choice of reflections for refinement. R-factors based on F^2^ are statistically about twice as large as those based on F, and R- factors based on ALL data will be even larger.

### Fractional atomic coordinates and isotropic or equivalent isotropic displacement parameters (Å^2^)

**Table:**

	*x*	*y*	*z*	*U*_iso_*/*U*_eq_	
N1	0.83203 (19)	0.0861 (6)	0.16111 (18)	0.0407 (6)	
N2	0.7398 (2)	0.0221 (8)	−0.0369 (2)	0.0498 (7)	
C1	0.8230 (2)	0.1476 (7)	0.0532 (2)	0.0399 (7)	
C2	0.9053 (3)	0.3372 (8)	0.0431 (3)	0.0489 (8)	
C3	0.9897 (3)	0.4449 (9)	0.1392 (3)	0.0508 (8)	
C4	0.9979 (3)	0.3777 (7)	0.2514 (3)	0.0459 (7)	
C5	0.9169 (2)	0.1971 (8)	0.2569 (3)	0.0429 (7)	
C6	1.0921 (3)	0.4935 (10)	0.3582 (3)	0.0653 (9)	
H6A	1.0781	0.4540	0.4251	0.098*	
H6B	1.1030	0.7269	0.3519	0.098*	
H6C	1.1555	0.3739	0.3662	0.098*	
O1	0.1957 (2)	−0.0891 (11)	0.1134 (2)	0.0995 (12)	
O2	0.2751 (3)	−0.0198 (10)	0.2954 (3)	0.0953 (11)	
O3	0.61995 (19)	0.6210 (7)	0.04604 (18)	0.0613 (7)	
O4	0.70099 (15)	0.7415 (6)	0.23282 (17)	0.0504 (5)	
N3	0.2718 (2)	0.0063 (7)	0.1991 (2)	0.0548 (7)	
C7	0.5306 (2)	0.4378 (8)	0.2704 (2)	0.0401 (6)	
C8	0.4456 (2)	0.2895 (8)	0.2828 (2)	0.0428 (6)	
C9	0.3641 (2)	0.1565 (7)	0.1870 (2)	0.0400 (6)	
C10	0.3648 (3)	0.1575 (8)	0.0790 (3)	0.0470 (7)	
C11	0.4522 (2)	0.3019 (8)	0.0683 (2)	0.0443 (7)	
C12	0.5345 (2)	0.4473 (7)	0.1622 (2)	0.0339 (5)	
C13	0.6260 (2)	0.6165 (7)	0.1462 (2)	0.0401 (7)	
H2A	0.896 (2)	0.396 (9)	−0.035 (3)	0.048 (8)*	
H3A	1.042 (3)	0.577 (9)	0.131 (3)	0.059 (10)*	
H5A	0.915 (3)	0.145 (9)	0.328 (3)	0.055 (9)*	
H7A	0.585 (2)	0.545 (7)	0.333 (2)	0.034 (7)*	
H9A	0.442 (3)	0.273 (8)	0.355 (4)	0.061 (10)*	
H10A	0.308 (3)	0.083 (10)	0.017 (3)	0.065 (11)*	
H11A	0.459 (3)	0.323 (10)	−0.006 (4)	0.070 (11)*	
H1N1	0.775 (4)	−0.035 (9)	0.171 (3)	0.059 (10)*	
H1N2	0.728 (3)	0.097 (9)	−0.107 (3)	0.052 (9)*	
H2N2	0.691 (3)	−0.087 (9)	−0.019 (3)	0.055 (10)*	

### Atomic displacement parameters (Å^2^)

**Table:**

	*U*^11^	*U*^22^	*U*^33^	*U*^12^	*U*^13^	*U*^23^
N1	0.0419 (14)	0.0442 (13)	0.0410 (12)	0.0003 (11)	0.0224 (11)	0.0046 (11)
N2	0.0518 (16)	0.0608 (17)	0.0392 (13)	0.0021 (13)	0.0217 (12)	0.0057 (12)
C1	0.0431 (16)	0.0395 (16)	0.0406 (14)	0.0106 (12)	0.0211 (13)	0.0076 (12)
C2	0.061 (2)	0.0446 (17)	0.0533 (17)	0.0047 (14)	0.0356 (16)	0.0118 (14)
C3	0.0485 (19)	0.0460 (18)	0.064 (2)	−0.0025 (15)	0.0299 (16)	0.0072 (15)
C4	0.0455 (17)	0.0389 (15)	0.0514 (16)	0.0041 (13)	0.0186 (14)	0.0031 (14)
C5	0.0444 (16)	0.0457 (16)	0.0393 (15)	0.0034 (13)	0.0183 (13)	0.0043 (13)
C6	0.052 (2)	0.064 (2)	0.066 (2)	−0.0083 (17)	0.0123 (16)	0.0025 (17)
O1	0.069 (2)	0.162 (3)	0.0674 (18)	−0.061 (2)	0.0293 (16)	−0.0244 (19)
O2	0.090 (2)	0.152 (3)	0.0620 (17)	−0.046 (2)	0.0490 (17)	−0.0059 (18)
O3	0.0633 (15)	0.0915 (18)	0.0378 (11)	−0.0184 (13)	0.0299 (11)	−0.0033 (12)
O4	0.0455 (12)	0.0696 (14)	0.0406 (11)	−0.0138 (11)	0.0224 (9)	−0.0070 (10)
N3	0.0509 (16)	0.0659 (18)	0.0539 (16)	−0.0126 (14)	0.0283 (13)	−0.0038 (14)
C7	0.0454 (16)	0.0462 (16)	0.0284 (12)	−0.0042 (13)	0.0151 (12)	−0.0026 (11)
C8	0.0498 (17)	0.0510 (18)	0.0330 (13)	−0.0057 (14)	0.0227 (12)	−0.0005 (12)
C9	0.0410 (16)	0.0405 (16)	0.0424 (15)	0.0001 (11)	0.0216 (13)	0.0031 (12)
C10	0.0476 (18)	0.0576 (19)	0.0325 (14)	−0.0095 (15)	0.0135 (13)	−0.0051 (13)
C11	0.0524 (18)	0.0542 (19)	0.0305 (13)	−0.0037 (14)	0.0216 (13)	−0.0002 (12)
C12	0.0377 (14)	0.0360 (14)	0.0297 (11)	0.0035 (10)	0.0160 (10)	0.0013 (10)
C13	0.0445 (17)	0.0470 (16)	0.0330 (14)	−0.0004 (13)	0.0203 (12)	0.0017 (11)

### Geometric parameters (Å, °)

**Table:**

N1—C1	1.347 (3)	O1—N3	1.208 (4)
N1—C5	1.355 (4)	O2—N3	1.210 (4)
N1—H1N1	0.97 (4)	O3—C13	1.241 (3)
N2—C1	1.328 (4)	O4—C13	1.251 (3)
N2—H1N2	0.89 (4)	N3—C9	1.466 (4)
N2—H2N2	0.90 (4)	C7—C8	1.374 (4)
C1—C2	1.410 (4)	C7—C12	1.399 (4)
C2—C3	1.350 (5)	C7—H7A	0.94 (3)
C2—H2A	0.97 (3)	C8—C9	1.366 (4)
C3—C4	1.408 (5)	C8—H9A	0.94 (4)
C3—H3A	0.94 (4)	C9—C10	1.375 (4)
C4—C5	1.352 (5)	C10—C11	1.387 (5)
C4—C6	1.499 (5)	C10—H10A	0.89 (4)
C5—H5A	0.93 (4)	C11—C12	1.379 (4)
C6—H6A	0.9600	C11—H11A	0.98 (4)
C6—H6B	0.9600	C12—C13	1.512 (4)
C6—H6C	0.9600		
			
C1—N1—C5	122.0 (3)	H6B—C6—H6C	109.5
C1—N1—H1N1	119 (2)	O1—N3—O2	122.2 (3)
C5—N1—H1N1	119 (2)	O1—N3—C9	119.3 (3)
C1—N2—H1N2	117 (2)	O2—N3—C9	118.4 (3)
C1—N2—H2N2	115 (2)	C8—C7—C12	120.6 (3)
H1N2—N2—H2N2	125 (3)	C8—C7—H7A	121.1 (18)
N2—C1—N1	119.0 (3)	C12—C7—H7A	118.2 (18)
N2—C1—C2	123.7 (3)	C9—C8—C7	118.7 (3)
N1—C1—C2	117.2 (3)	C9—C8—H9A	118 (2)
C3—C2—C1	120.2 (3)	C7—C8—H9A	123 (2)
C3—C2—H2A	122.6 (19)	C8—C9—C10	122.8 (3)
C1—C2—H2A	117.1 (19)	C8—C9—N3	118.9 (2)
C2—C3—C4	121.7 (3)	C10—C9—N3	118.3 (3)
C2—C3—H3A	119 (2)	C9—C10—C11	117.9 (3)
C4—C3—H3A	119 (2)	C9—C10—H10A	120 (2)
C5—C4—C3	116.0 (3)	C11—C10—H10A	122 (2)
C5—C4—C6	122.1 (3)	C12—C11—C10	121.1 (3)
C3—C4—C6	121.8 (3)	C12—C11—H11A	115 (2)
C4—C5—N1	122.8 (3)	C10—C11—H11A	124 (2)
C4—C5—H5A	122 (2)	C11—C12—C7	118.9 (3)
N1—C5—H5A	116 (2)	C11—C12—C13	119.7 (2)
C4—C6—H6A	109.5	C7—C12—C13	121.4 (2)
C4—C6—H6B	109.5	O3—C13—O4	124.8 (3)
H6A—C6—H6B	109.5	O3—C13—C12	116.3 (2)
C4—C6—H6C	109.5	O4—C13—C12	118.8 (2)
H6A—C6—H6C	109.5		
			
C5—N1—C1—N2	177.7 (3)	O2—N3—C9—C8	−6.1 (5)
C5—N1—C1—C2	−0.7 (4)	O1—N3—C9—C10	−5.5 (5)
N2—C1—C2—C3	−177.3 (3)	O2—N3—C9—C10	173.9 (3)
N1—C1—C2—C3	1.1 (4)	C8—C9—C10—C11	−0.3 (5)
C1—C2—C3—C4	−1.0 (5)	N3—C9—C10—C11	179.6 (3)
C2—C3—C4—C5	0.6 (5)	C9—C10—C11—C12	−1.6 (5)
C2—C3—C4—C6	179.3 (3)	C10—C11—C12—C7	2.1 (4)
C3—C4—C5—N1	−0.2 (5)	C10—C11—C12—C13	−177.2 (3)
C6—C4—C5—N1	−178.9 (3)	C8—C7—C12—C11	−0.8 (4)
C1—N1—C5—C4	0.3 (4)	C8—C7—C12—C13	178.5 (3)
C12—C7—C8—C9	−1.1 (4)	C11—C12—C13—O3	1.7 (4)
C7—C8—C9—C10	1.6 (5)	C7—C12—C13—O3	−177.6 (3)
C7—C8—C9—N3	−178.3 (3)	C11—C12—C13—O4	−178.7 (3)
O1—N3—C9—C8	174.4 (4)	C7—C12—C13—O4	2.1 (4)

### Hydrogen-bond geometry (Å, °)

**Table:**

*D*—H···*A*	*D*—H	H···*A*	*D*···*A*	*D*—H···*A*
N1—H1N1···O3^i^	0.97 (5)	2.47 (4)	3.238 (5)	136 (3)
N1—H1N1···O4^i^	0.97 (5)	1.77 (5)	2.711 (4)	163 (3)
N2—H1N2···O4^ii^	0.89 (4)	2.02 (4)	2.905 (4)	179 (5)
N2—H2N2···O3^i^	0.91 (4)	1.92 (4)	2.804 (5)	165 (4)
C3—H3A···O1^iii^	0.93 (4)	2.58 (4)	3.514 (6)	176 (3)

Symmetry codes: (i) *x*, *y*−1, *z*; (ii) *x*, −*y*+1, *z*−1/2; (iii) *x*+1, *y*+1, *z*.
